# Structural determinants of peanut-induced anaphylaxis

**DOI:** 10.1016/j.jaci.2024.12.1095

**Published:** 2025-01-11

**Authors:** Scott A. Smith, Rebecca A. Shrem, Bruno B. C. Lança, Jian Zhang, Joyce J. W. Wong, Derek Croote, R. Stokes Peebles, Benjamin W. Spiller

**Affiliations:** a Department of Medicine, Vanderbilt University Medical Center, Nashville; b Department of Pathology, Vanderbilt University Medical Center, Nashville; c Department of Pharmacology, Vanderbilt University School of Medicine, Vanderbilt University, Nashville; d IgGenix Inc, San Francisco

**Keywords:** IgE, mAb, epitope, allergy, anaphylaxis

## Abstract

**Background:**

Human IgE mAbs recognizing peanut allergens have recently become available, but we lack a detailed understanding of how these IgEs target allergens.

**Objective:**

We sought to determine the molecular details of the antibody-allergen interaction for a panel of clinically important human IgE mAbs and to develop strategies to disrupt disease causing antibody-allergen interactions.

**Methods:**

We identified candidates from a panel of epitope binned human IgE mAbs that recognize 2 important and homologous peanut allergens, Ara h 2 and Ara h 6. Crystal structures were determined revealing the interfaces (antigenic sites) of exemplars of 5 common IgE bins.

**Results:**

Among the common antigenic sites on Ara h 2 and Ara h 6, 2 sites (A and B) are highly conserved between the allergens, explaining the cross-reactivity of antibodies that recognize these sites. Three sites (C, D, and F) involve residues that are not conserved between the allergens. Of the 5 common sites, 3 sites (B, C, and D) involve residues that are near each other only when the allergens are properly folded, such that these sites are conformational. Two additional sites (sites A and F) involve largely linear stretches of amino acids. Site F targeted antibody, 38B7, binds to a peptide sequence DPYSP^OH^S, in which hydroxylation of the last proline is critical for binding. This sequence is repeated 2 or 3 times depending on the Ara h 2 isoform, enabling 38B7 to induce anaphylaxis as a single mAb, without a second antibody. We have mutated key residues in each site and created a panel of hypoallergens, having reduced IgE mAb binding and lacking the ability to induce anaphylaxis in our murine model.

**Conclusion:**

We created a structural map of the IgE antibody response to the most important peanut allergen proteins to enable the design of new allergy immunotherapies and vaccines. (J Allergy Clin Immunol 2025;155:1547–56.)

Food allergy has become common in the United States with 7.6% of children and 10.8% of adults affected.^1,2^ Rates of food allergy have increased remarkably in recent decades with a corresponding increase in severe reactions, such as those requiring hospitalization.^3^ Fatal anaphylaxis in both the United States and the United Kingdom is mostly caused by peanut or tree nut allergies and occurs largely in patients known to have nut allergy.^4^ In both peanuts and tree nuts, the 2S albumin protein family are important allergens.^5^ They are among the most important nut allergens clinically, and anti–Ara h 2 and anti–Ara h 6 IgE antibodies are the best predicters of peanut allergy severity.^6–8^ A molecular understanding of how IgE antibodies against these important allergens cause disease requires identification of the molecular epitopes and therefore requires human monoclonal IgE clones or human IgG clones that recognize the same epitopes. Recently, studies have developed human IgEs against peanut allergens and have identified a critically important linear epitope on Ara h 2.^9,10^ In addition, 2 groups have shown that a subpopulation of human IgG memory B cells (mostly IgG_1_ and IgG_4_) are poised to switch to IgE, suggesting that clones within the IgG pool ultimately give rise to IgE-secreting B cells.^11,12^ In fact, studies of IgG_4_ mAbs developed from subjects following successful immunotherapy that are capable of blocking IgE binding confirm that at least some of the clones in the IgG pool are highly relevant for allergy because they recognize Ara h 2 at similar epitopes as IgE.^13–15^ Taken together, these studies reveal that both allergy-causing IgE and protective immunotherapy expanded IgG clones arise from the same allergen-specific IgG memory B-cell repertoire.

Despite recent progress, a molecular understanding of IgE interactions with disease-causing epitopes remains necessary to fully understand allergic sensitivity. Key questions are which epitopes are critical and why certain epitopes and pairs of epitopes potently cross-link and activate the high-affinity IgE receptor (FcεRI), causing degranulation and mediator release. Atomic structures of allergen-antibody complexes are needed to address these questions. Three such structures with IgGs have been reported, and these structures have enabled the beginning of efforts to create mutant proteins, termed hypoallergens, that may prove useful as therapeutics.^13,14^

Our companion article^16^ describes a panel of human IgE mAbs identified from patients with allergy. These IgE mAbs were shown to form 5 epitope bins, where each bin is a subset of mAbs that compete with each other for binding to the allergen, but do not compete with mAbs from other bins. In the current article, we show that these bins are discrete molecular surfaces, and we present atomic structures of these disease-causing antibodies in complex with allergens and identify the molecular epitopes of 5 antibody bins. We identify Ara h 2– and Ara h 6–specific and cross-reactive epitopes as well as linear and conformational epitopes. With structures in hand, we have developed a panel of hypoallergens and show that they prevent anaphylaxis in a sensitized mouse model. Finally, we show that the atomic structure of 38B7 bound to Ara h 2 and a peptide derived from a repetitive sequence on a loop within Ara h 2 reveals a key role for proline hydroxylation in peanut allergy.

## METHODS

### Expression and purification of IgGs, antigen-binding fragments, and allergens

Allergen proteins used in crystallization experiments were purified from ExpiCHO cells (Thermo FIsher Scientific, Waltham, Mass). Allergens were truncated at the amino terminus, hexahistidine tagged at the carboxy terminus, codon optimized, and cloned into pcDNA3.4 (GenScript, Piscataway, NJ). Ara h 2c lacks the first 4 residues, and Ara h 6c lacks the first 12 residues. Other mutants of Ara h 2 or Ara h 6 were expressed in expi293 (Thermo Fisher) or expiCHO cells. Allergens were purified by Talon affinity chromatography (Takara Bio USA, San Jose, Calif), concentrated to 2 mg/mL and stored at −80°C. 16A8, 38B7, 1H9, and 8F3 antigen-binding fragments (Fabs) were made by papain digestion (Worthington Enzymes, Lakewood, NJ) of purified IgG^16^ and purified over an anti-CH1 column (Thermo Fisher Scientific). 13D9 was synthesized as an Fab and cloned into pcDNA3.4 (GenScript) and purified over an anti-CH1 column (Thermo Fisher Scientific). Antibodies and Fabs were concentrated to 10 mg/mL and stored at −80°C.

### Mouse studies

Human FCεRI transgenic mice [B6.Cg-Fcεr1atm1KntTg(F-CER1A)1Bhk/J] were purchased from The Jackson Laboratory (Bar Harbor, Me) (stock 010506), bred and genotyped to verify that they carry both human FCER1A with the human FCER1A promoter and a disrupted Fcεr1atm1Knt, which blocks expression of murine FCER1A.^17^ Transgenic mice (hemizygous for the transgene and homozygous for targeted deletion of mouse FcεRI) were sensitized by intravenous injection of IgE (100 μg total) and challenged by intraperitoneal injection of 50 μg purified recombinant allergen or 0.5 mL of 15% peanut extract (ALK-Abelló, Horsholm, Denmark) in PBS. Core body temperature was monitored over 90 minutes using implanted temperature probes. Mouse studies were carried out in accordance with recommendations in the National Institutes of Health *Guide for the Care and Use of Laboratory Animals*.

### Statistics

Statistical analysis was performed using Prism 9.2 (GraphPad Software). Error bars represent SD.

### Crystallization

Fabs and proteins or peptides were mixed, and the appropriate complexes were isolated by gel filtration on a Superdex 200 (Cytiva, Malborough, Mass) column equilibrated with 150 mM sodium chloride (Sigma,Burlington, Mass), 10 mM Tris pH 7.5 (Sigma). For the Ara h 6c-16A8 complex, 1.2-fold excess 16A8 was used. For the remaining Fab complexes, allergen and a single Fab were added in equivalent stoichiometry, and 1.2-fold excess of the second Fab was used. For Ara h 2c-8F3–38B7, 38B7 was in excess. For Ara h 6c-8F3–1H9, 1H9 was in excess. For Ara h 6c-13D9–16A8, 13D9 was in excess. The complex was concentrated to approximately 15 mg/mL, and crystallization trials were performed by hanging drop vapor diffusion. Initial hits were optimized, and crystals were cryoprotected and flash-cooled in liquid nitrogen. 16A8-Ara h 6c crystals were grown from a reservoir containing 100 mM Bis Tris (Sigma) pH 5.5, 100 mM ammonium sulfate (Sigma), and 20% polyethylene glycol (PEG) 3350 (Sigma). These crystals were cryocooled directly from well solution. 13D9–16A8-Ara h 6c crystals were grown from a reservoir containing 100 mM Bis Tris pH 5.25 (Sigma), 75 mM ammonium acetate (Sigma), and 16% PEG 10,000 (Sigma) and were cryoprotected with 20% glycerol (Sigma). 8F3–1H9-Ara h 6c crystals were grown from a reservoir containing 240 mM sodium malonate pH 7.0, 20% PEG 3350 (Sigma), and cryoprotected with 10% glycerol (Sigma). 8F3–38B7-Ara h 2c crystals were grown from a reservoir containing 21% PEG 8000 (Sigma), 200 mM sodium chloride (Sigma), 50 mM sodium phosphate (Sigma), and 50 mM citrate pH 4.2 (Sigma) and were cryoprotected with 20% glycerol (Sigma). 38B7-peptide crystals were grown from a reservoir containing 100 mM sodium citrate pH 5.6, 20% isopropyl alcohol (Sigma), 20% PEG 4000 (Sigma), and 10 mM cadmium chloride (Sigma) and were cryoprotected with 10% glycerol. 38B7 crystals were grown from a reservoir containing 20% PEG 4000 and 100 mM 2-(*N*-morpholino)ethanesulfonic acid pH 6.5 (Sigma) and were cryoprotected with 20% glycerol (Sigma).

### Data collection and structure determination

Diffraction data were collected at LS-CAT Sector 21 at the Advance Photon Source (Argonne, Ill). All data were collected from single crystals maintained in a gaseous nitrogen stream at 100K. Data were indexed, integrated, and scaled with XDS and Aimless as called from AutoProc.^18–20^ Data collection statistics are given in Table E1 (in the Online Repository available at www.jacionline.org). Molecular replacement was performed with Molrep^21^ by iteratively searching a library of approximately 200 Fab fragments. Solutions were verified with Phaser, and initial models were improved by automated rebuilding using AutoBuild in Phenix.^22^ In no case could either Ara h 2c or Ara h 6c be readily located by molecular replacement, and both were manually built. Resulting models were refined in Phenix.^22^ Refinement included rigid body refinement of the individual domains, simulated annealing, positional refinement, individual B-factor refinement, and TLS refinement.^23^ Models were rebuilt and side chains added in COOT using simulated annealing composite omit maps generated with Phenix to help rebuild challenging areas.^22,24,25^ The structures have been deposited in the Protein Data Bank under accession codes 8SHV, 8SHW, 8SJ6, 8SI1, 8SJ4, and 8SJA.

### Peptide binding and inhibition ELISA

IgE mAb 38B7 binding to hydroxylated and nonhydroxylated DPYSPS containing peptide (EDSYERDPYSP^OH^SQD) (Genscript) was evaluated both directly and indirectly using ELISA. In direct binding analysis, hydroxylated and nonhydroxylated DPYSPS, random control peptide, rAra h 2.01, or rAra h 2 with DPYSPS motif deleted (residues 39–60 and 39–71) was used to coat ELISA plates. After ELISA plate blocking, a halving dilution series of 38B7 IgE in blocking solution then was added, starting at a concentration of 10 μg/mL. After washing, secondary antibody (mouse anti-human IgE Fc, 9160–05; Southern Biotech) was applied at a 1:1000 dilution in blocking solution. Finally, after washing, ultra-TMB substrate solution (34029; Thermo Fisher Scientific) was added at 25 μL/well, before reading at OD 450 nm. For inhibition assays, binding of 38B7 to rAra h 2.01, rAra h 2.02, and nAra h 2 was inhibited by 100× molar excess of hydroxylated, nonhydroxylated EDSYERDPYSPSQD, or random peptide control added before addition of a halving dilution series of 38B7 IgE in blocking solution. After washing, secondary antibody was applied at a 1:1000 dilution in blocking solution. Again, ultra-TMB substrate solution was added at 25 μL/well, before reading at OD 450 nm.

### IgE mAb kinetic analysis

Kinetic parameters of 38B7 and 16A8 antibody binding to Ara h 2 variants and peptide epitopes were measured using a Carterra LSA instrument. 38B7 and 16A8 binding to rAra h 2_loopdel, Random_std, and rAra h 6; 38B7 binding to 16A8pep_std; and 16A8 binding to rAra h 2.02 and 38B7pep_OH were measured using antibodies noncovalently bound to PAGHC30M (Protein A/G derivatized linear polycarboxylate hydrogel, medium charge density, 30-nm coating thickness) sensor chips using HBS-TE with the LSA multichannel printhead. Antibodies were coupled to the surface at 0.1, 0.3, 1.0, 3.0, 10.0, and 30.0 μg/mL concentration in HBS-TE (Carterra, Salt Lake City, Utah), 0.05% Tween-20 (Carterra) for 10 minutes. This was followed by 8 injections of buffer, then serial injections of analyte at 0.16, 0.8, 4, 20, 100, and 500 nM concentration using the LSA single flow cell over the antibody-coupled chip in HBS-TE, 0.05% BSA running buffer (Carterra), each with 5 minutes association time and 15 minutes of dissociation time. The chip is regenerated with 2 × 60 seconds injections of 10 mM glycine pH 2.0 for subsequent cycles of antibody functionalization and binding to analytes.

## RESULTS

In this article, we have focused on determining antigenic sites for IgEs targeting the most clinically important allergens, Ara h 2 and Ara h 6.^6–8^ The IgEs that target epitopes on these antigens are described comprehensively in our companion article^16^ and shown to compete with serum IgE both by blocking serum IgE binding to peanut allergens and by reducing response in a skin prick test. A total of 6 epitope bins are described: antibodies in 2 epitope bins, sites A and B, recognize both Ara h 2 and Ara h 6; 3 bins, sites C, D, and E, are Ara h 6 specific; and 1 bin, site F, is Ara h 2 specific. We have used x-ray crystallography using recombinant proteins to determine crystal structures of representative members of bins A, B, C, D, and F. We determined crystal structures (Table E1) of Fab fragments of the prototypical antibodies bound to Ara h 2c (lacking the first 4 residues) or Ara h 6c (lacking the first 12 residues).

**FIG 1.**
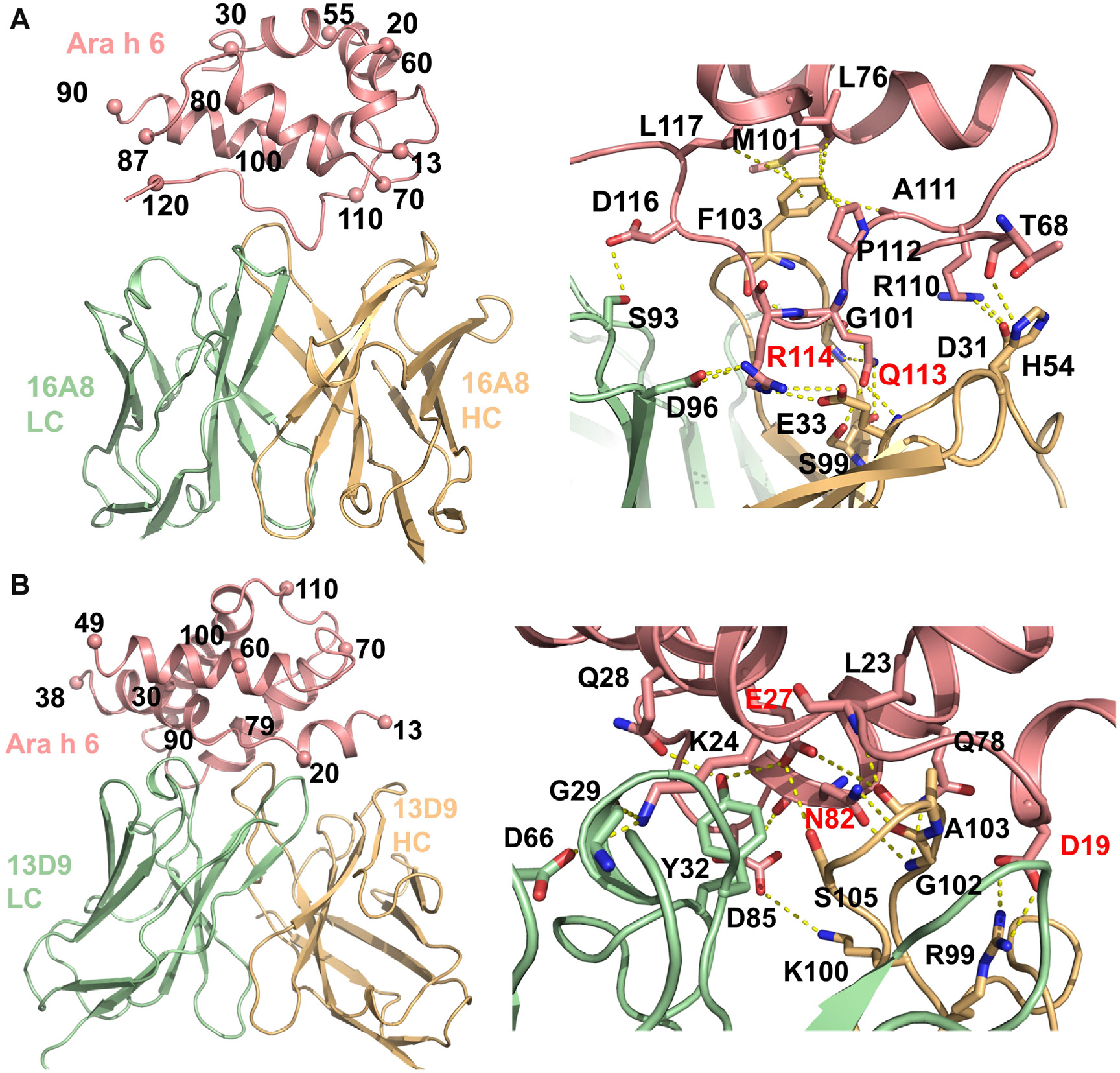
Structures of cross-reactive clones, 16A8 and 13D9. Sequential numbering, excluding secretion signals, are used for all proteins. Allergens are colored *salmon*, HCs are *light orange*, and LCs are *light green*. Only variable regions of the Fabs are shown. The *left panels* show ribbon diagrams of each complex. Ara h 6c is numbered approximately every 10 residues. Residues mutated in hypoallergens are labeled in *red*. **(A)** Site A is largely formed by the last approximately 10 residues of Ara h 6c *(right)*, which lie in a groove across both HCs and LCs of 16A8. Critical residues in site A, R114 and Q113, are shown in *red*. These residues make salt bridges from E33_LC_ and D96_HC_ to R114 and H-bonds between Q113 to S99_HC_ and G101_HC_. Additionally, R110 forms a salt bridge with D31_HC_, and D116 and T68 H-bond with S93_LC_ and H54_HC_, respectively. F103_HC_ fits into a hydrophobic pocket on Ara h 6c made by L77, M101, A111, P112, and L117. **(B)** 13D9 recognizes site B in Ara h 6c using both HCs and LCs. Residues 13 to 24 of Ara h 6c, from the first 2 helices and the intervening loop, form a continuous epitope for 13D9, but additional contacts are made to helix 4, just below residue 79 in the *left panel*. The *right panel* shows key salt bridges and electrostatic interactions. Of these key residues (labeled *red*), E27 forms H-bonds with Y32_LC_, S105_HC_, and A103_HC_; N82 forms 2 H-bonds with G102_HC_ and 1 with Q50_LC_ (main chain of N82 to side chain of Q50 [not visible]); and D19 forms a salt bridge with R99_HC_. Additional, observable interactions are Q28 H-bonds to Y32_LC_, K24 makes a salt bridge with D66_LC_ and H-bonds to G29_HC_, D85 makes a salt bridge with K100_HC_, L23 H-bonds to A103_HC_, and Q78 H-bonds to G102_HC_.

Two cross-reactive sites, sites A and B were recognized by 12 and 8 clones, respectively.^16^ Here we focus on clones 16A8 and 13D9 as prototypical members of bins A and B, as shown in Fig 1. Site A includes approximately 720 Å^2^ of solvent accessible surface on Ara h 6c and is centered on Gln 113 and Arg 114, which are recognized by multiple salt bridges and hydrogen bonds (H-bonds) (Fig 1, A). R114 is recognized by salt bridges to light chain (LC) of D96 and E33_LC_ and is pinned between complementarity determining region L3 (CDRL3) and CDRH1 of 16A8. Q113 forms H-bonds to E33, S99, and G101. 16A8 largely recognizes a linear epitope consisting of Ara h 6 residues 110 to 117, which is conserved in Ara h 2 (Fig 1, A). Although the recognition site is largely a linear peptide, 16A8 inserts heavy chain (HC) of F103 into a hydrophobic pocket formed by M101, L76, L117, and P112 of Ara h 6c. This interaction can exist only with the folded conformation of Ara h 6c.

Site B involves both a linear sequence involving residues 15 to 27 of Ara h 6c, which comprise the first 2 helices of Ara h 6 and the intervening loop, as well as helix 4 to which 13D9 forms salt bridges and H-bonds (Fig 1, B). Recognition of the linear sequence or Ara h 6c involves salt bridges between D19 and R99_HC_ as well as K24 and D66_LC_. Two highly coordinated residues form the center of this interface. E27 makes H-bonds to Y32_LC_, S105_HC_, and A103_HC_, and N82 forms 2 H-bonds to G1022_HC_. Binding of 13D9 buries approximately 930 Å^2^ of Ara h 6c, and in contrast to 16A8, 13D9 does not recognize peptides from Ara h 2 or Ara h 6, differing from some site B directed antibodies (Fig 1).^16^

Two Ara h 6-specific sites, sites C and D, are also conformational, as shown in Fig 2. 1H9 recognizes site C by binding the carboxy terminal end of helix 4, the amino terminus of helix 5, the loop between helices 4 and 5, and the carboxy terminus of Ara h 6c (Fig 2, A). The sequences form a continuous interface only in the context of a folded Ara h 6, and these sequences are not conserved in Ara h 2, making site C both Ara h 6 specific and conformationally specific. The interface involves exclusion of solvent from approximately 910 Å^2^ of Ara h 6c and is roughly centered on R90 and Q91 (Fig 2, A). R90 is highly coordinated, forming a salt bridge with D95_HC_ and a side chain H-bond to N35_HC_, as well as H-bonds to the main chains of N92_LC_ and S95_LC_. Q91 is packed between G33_HC_ and N52_HC_ and forms H-bonds with T30, G33, and T53.

Site D, as defined by 8F3, is also a conformational epitope, burying approximately 750 Å^2^ of Ara h 6c and involving residues from helices 2, 3, and 5 of Ara h 6c (Fig 2, B). Site D residues R33, Q55, E99, and Q106 are recognized by the HC of 8F3. These residues are buried in the complex and involved in multiple polar interactions. R33 forms a salt bridge with D102_HC_ and H-bonds with S33_HC_ and Y54_HC_. S33_HC_ also forms an H-bond with S102. Q55 forms hydrogen bonds with R107_HC_ and D102_HC_. E99 H-bonds with Y54_HC_, and Q106 hydrogen bonds with S33_HC_ (Fig 2, B). Two peripheral HC interactions include a salt bridge and H-bond between R98 and D30 and an H-bond between the indole nitrogen of W105_HC_ and the main chain of Q105. Finally, a single LC interaction is formed between D53 and the main chain of S92_LC_.

**FIG 3.**
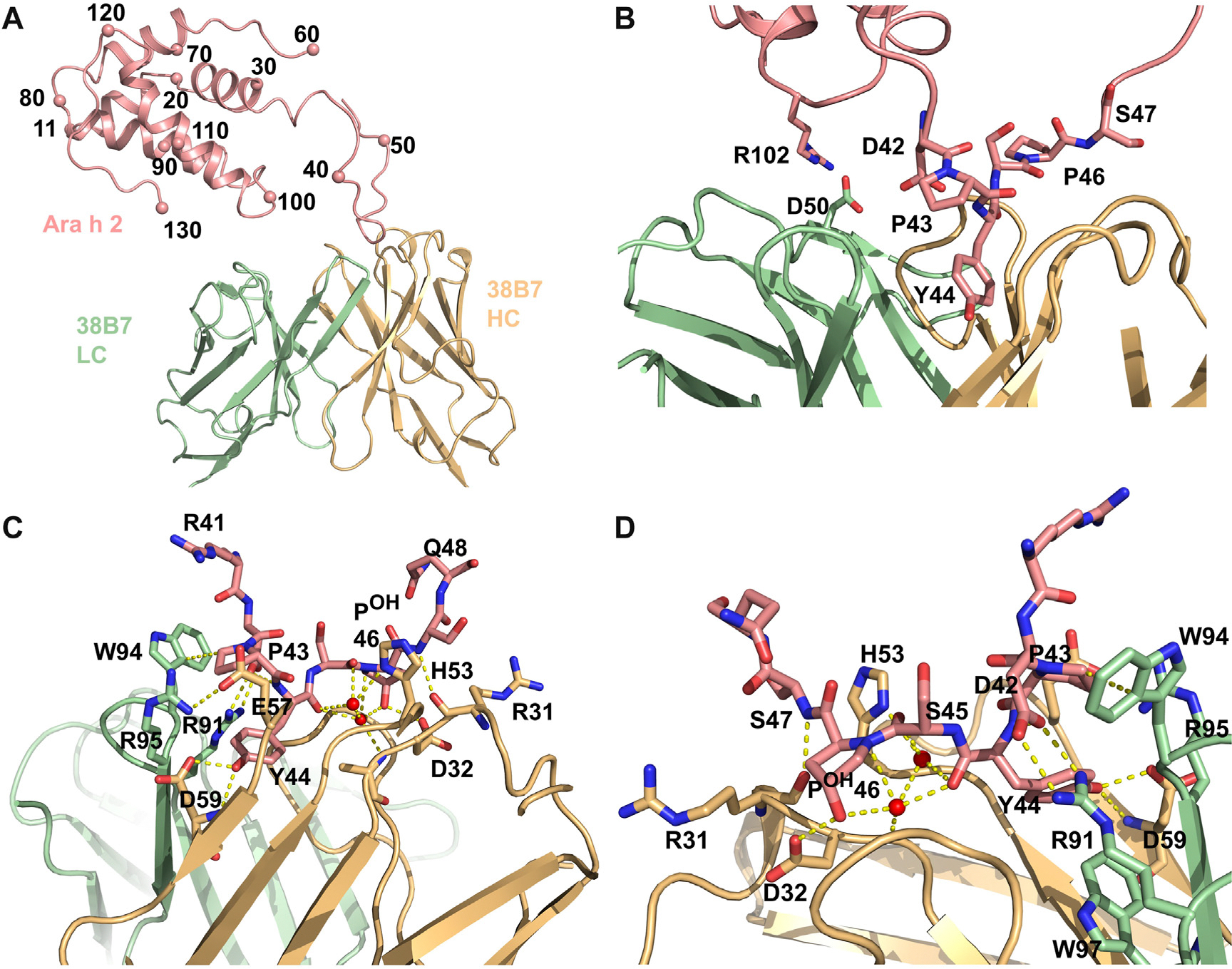
Structures of Ara h 2 specific, DPYSP^OH^S targeted, 38B7. Allergens are colored *salmon*, HCs are *light orange*, and LCs are *light green*. **(A** and **B)** A 1:1 complex of Ara h 2c and 38B7 Fab. The DPYSPS repeat containing loop is bound between CD3 and CDRs 1 and 2 of the HC, and R102 is recognized by D50 in CDRL2. The low resolution of the 38B7-Ara h 2c structure *(A* and *B)* prevents most side chain assignments. R102 of Ara h 2c and D50_HC_ of 38B7 are in proximity and inferred to be involved in a salt bridge. **(B** and **C)** Two views, approximately 1808 rotated along a vertical axis, of the DPYSP^OH^S-38B7 complex. The 38B7 bound structures of Ara h 2c *(B)* and the hydroxyproline-containing peptide *(C)* are similar. Within the DPYSPS motif, every residue is recognized: D42 salt bridges with R91_LC_; P43 packs against W94_LC_; Y44 is buried and forms 2 H-bonds with D59_HC_; S44 faces solvent and H-bonds to an ordered water, which also H-bonds to H53_LC_; HYP46 forms multiple interactions. The hydroxyl group of HYP46 H-bonds to D32_HC_ and to a water that bridges through another H-bond to the main chain carbonyl of Y44, stabilizing the bound conformation of the peptide. S47 forms a main chain H-bond with R31_HC._

**FIG 4.**
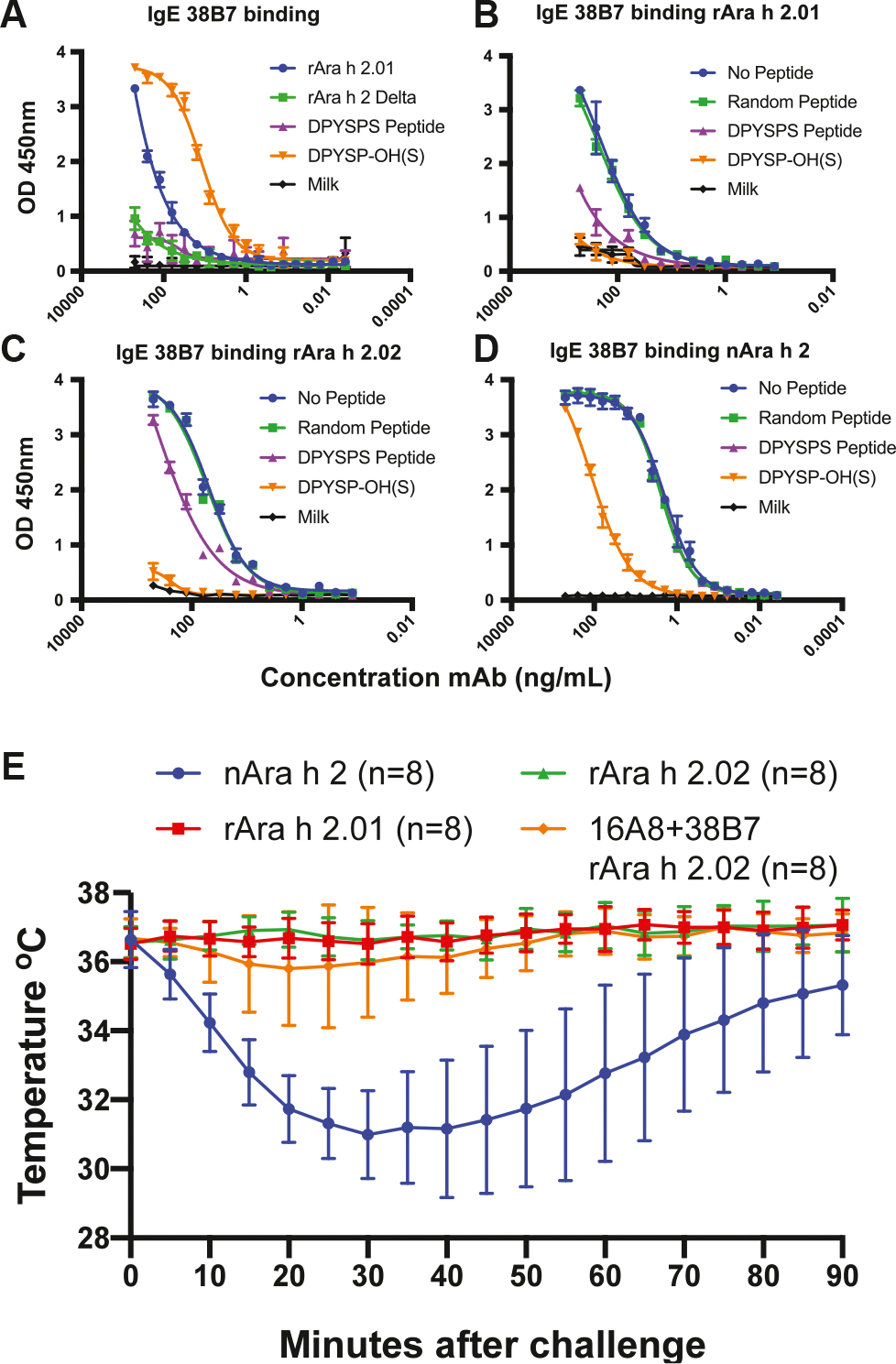
Antibody 38B7 binds repeating hydroxylated DPYSPS motif of Ara h 2. **(A)** IgE mAb binding to hydroxylated and nonhydroxylated DPYSPS-containing peptide, rAra h 2.01, and rAra h 2 with the DPYSPS-containing loop deleted (residues 39–60). Inhibition of binding of 38B7 to **(B)** rAra h 2.01, **(C)** rAra h 2.02, and **(D)** nAra h 2 by hydroxylated and nonhydroxylated DPYSPS-containing peptide. **(E)** Passive systemic anaphylaxis following sensitization with IgE 38B7 and challenge with nAra h 2, rAra h 2.01, and rAra h 2.02 (mice also sensitized with 16A8 and 38B7). Anaphylaxis was monitored using an implanted temperature probe for 90 minutes following challenge. Time points with calculated *P* values < .05 are underscored with a colored bar. Data are means ± SDs of each experimental group. The number of mice (n) for each experimental group is shown.

Site F is unique, in that 38B7 can mediate anaphylaxis without a second IgE clone.^16^ The 38B7-Ara h 2c structure reveals that 38B7_HC_ binds a linear epitope (DPYSPS), whereas the LC likely forms a single salt bridge to R102 on helix 5. Importantly, the second proline in the DPYSPS motif is hydroxylated in native (*Arachis hypogaea* produced) Ara h 2 (nAra h 2),^26–28^ and a peptide containing this modification binds slightly tighter than HEK cell–produced rAra h 2.01 and much tighter than the same peptide without the modification (Table I). A high-resolution structure of this peptide bound to 38B7 (Fig 3; Table E1) reveals that the hydroxyl group on hydroxyproline (P^OH^46) is directly recognized by an H-bond to 38B7 (HC residue D32) and forms a water-mediated H-bond with the main chain carbonyl of Y44. Despite tight binding (dissociation equilibrium constant = 20 nM) (Fig 4, Table I), the 38B7-peptide complex buries only approximately 410 Å^2^ of Ara h 2 in the peptide complex and approximately 610 Å^2^ in the Ara h 2c interface. As shown in Fig 3, DPSYP^OH^S peptide is bound between CDR3 and CDRs 1 and 2 of the HC. Two water molecules are highly coordinated by the peptide, one of which H-bonds to the proline hydroxyl group and to the main chain carbonyl of Y44, stabilizing the bound conformation. The side chain of Y44 is completely buried in the complex. In addition to these interactions, R102 of Ara h 2c and D50_HC_ of 38B7 are in proximity and inferred to be involved in a salt bridge.

**FIG 5.**
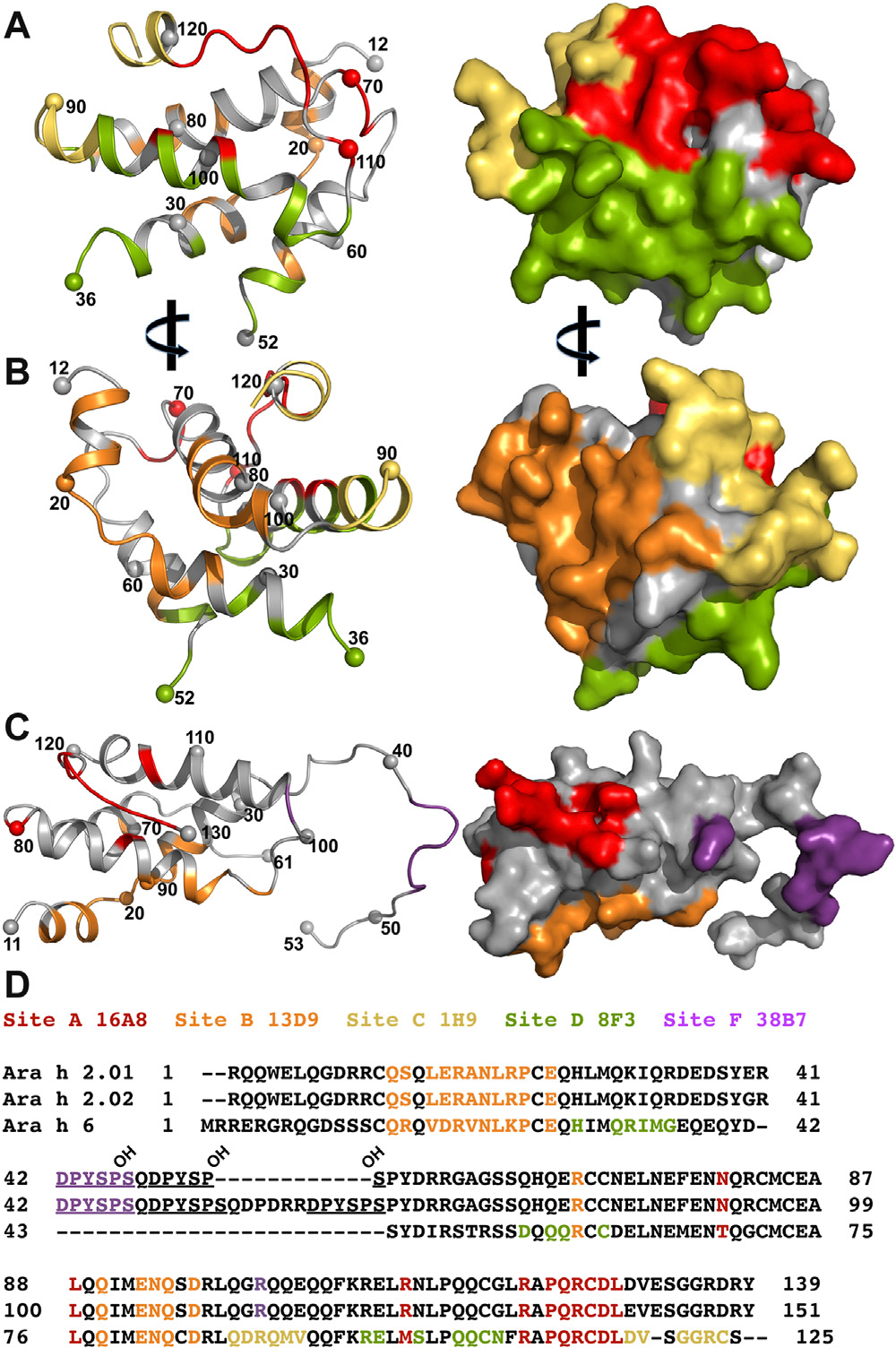
Molecular footprints of the 4 major antigenic sites on Ara h 2c and Ara h 6c. **(A)** Ribbon diagram *(left)* and molecular surface of Ara h 6c showing the surfaces buried from solvent in 16A8 *(red)*, 13D9 *(orange)*, 1H9 *(yellow)*, and 8F3 *(green)*. Residues not recognized by antibodies are shown in *gray*. **(B)** As in *(A)*, but rotated 1808 from the view in *(A)*. **(C)** Ribbon diagram *(left)* and molecular surface showing, in *dark purple*, the surface on Ara h 2 buried in the 38B7 interface. Critical residues for interactions in *(A-D)* are shown in **(D)** a sequence alignment of Ara h 2.01, Ara h 2.02, and Ara h 6. Contact residues are color coded as in *(A* and *B)*. Hydroxylated prolines are indicated.

Fig 5 summarizes the structural findings and shows the epitope footprints on Ara h 2 and Ara h 6. Ara h 2 is recognized by site A, B, and F targeted antibodies, whereas Ara h 6 is recognized by site A, B, C, and D targeted antibodies. These sites are non-overlapping and cover nearly all of Ara h 6. 1H9 and 8F3 bind sites that are largely conserved in Ara h 2, but the small number of differences between the allergens within the epitope (Fig 5, D) significantly reduce binding, suggesting that cross-reactive antibodies to these sites could be made. Site F, by contrast, is formed by a repeating peptide that is not found in Ara h 6.

The DPYSPS motif appears twice in Ara h 2 isoform 2.01 and 3 times in isoform 2.02 (Fig 5, D), explaining why natural peanut extract, containing approximately equal molar ratio of the isoforms, with hydroxylated prolines, is such a potent trigger for anaphylaxis in our mouse model (Fig 4, E). As shown in Fig 4 and Table I, this motif is bound tightly when hydroxylated. The hydroxylated peptide is bound approximately 350-fold more tightly than the nonhydroxylated peptide, and nAra h 2 is bound approximately 30-fold more tightly than rAra h 2.01 (Table I). Similarly, the hydroxylated peptide potently blocks rAra h 2 binding, but not nAra h 2 (Fig 4, A–D). As shown in Fig 4, E, a significant decrease in temperature occurred after mice were sensitized with IgE 38B7 and challenged with natural Ara h 2, but not when challenged with either rAra h 2.01 or rAra h 2.02. Three Fabs can bind Ara h 2.02 simultaneously, but it is not clear if this would require 2 or 3 IgEs to provide the Fabs. In either case, binding to IgEs that are prebound to FCεR1 on a mast cell would likely cross-link more receptors than a site A and B complex, or a similar complex.

With molecular structures in hand, we set out to disrupt key sites of recognition of Ara h 6c, with the long-term goal of creating reagents that can be used for immunotherapy or immunization. Before use, it will be necessary to evaluate safety and to demonstrate that such reagents lead to a regulatory immune response. Residues forming multiple interactions with antibodies and typically located near the center of each interface were targeted for mutation. The mutations made were as follows: Q113R and R114E for site A; D19R, E27A, and N82A for site B; R90Q and Q91A for site C; and Q33E, Q55A, E99R, and Q106E for site D (colored red in Figs 1 and 2). Mutations to sites C and D were also combined in a protein (Mut CD). As noted above, these residues all are highly coordinated in the complex. In site A, Arg 114 forms 2 salt bridges, and Gln 113 forms 3 H-bonds (Fig 1, B). In site B, D19, E27, and N82 form a total of 5 H-bonds and a salt bridge (Fig 1, C). In site C, R90 and Q91 are similarly highly coordinated with 5 H-bonds with appropriate geometry, a salt bridge, and a hydrophobic interaction to the aliphatic portion of R90 (Fig 2, B). In site D, no single or pair of residues appeared to be essential for the interaction, and 4 mutations were made: Q33E, Q55A, E99R, and Q106E. These mutations led to introduction of 2 new negative charges (Q33E and Q106E) and a charge reversal (E99R) (Fig 2, D). Finally, site F required no engineering to create a hypoallergen because proline hydroxylation is essential for potent 38B7 binding and anaphylaxis (Figs 3 and 4; Table I).

**FIG 6.**
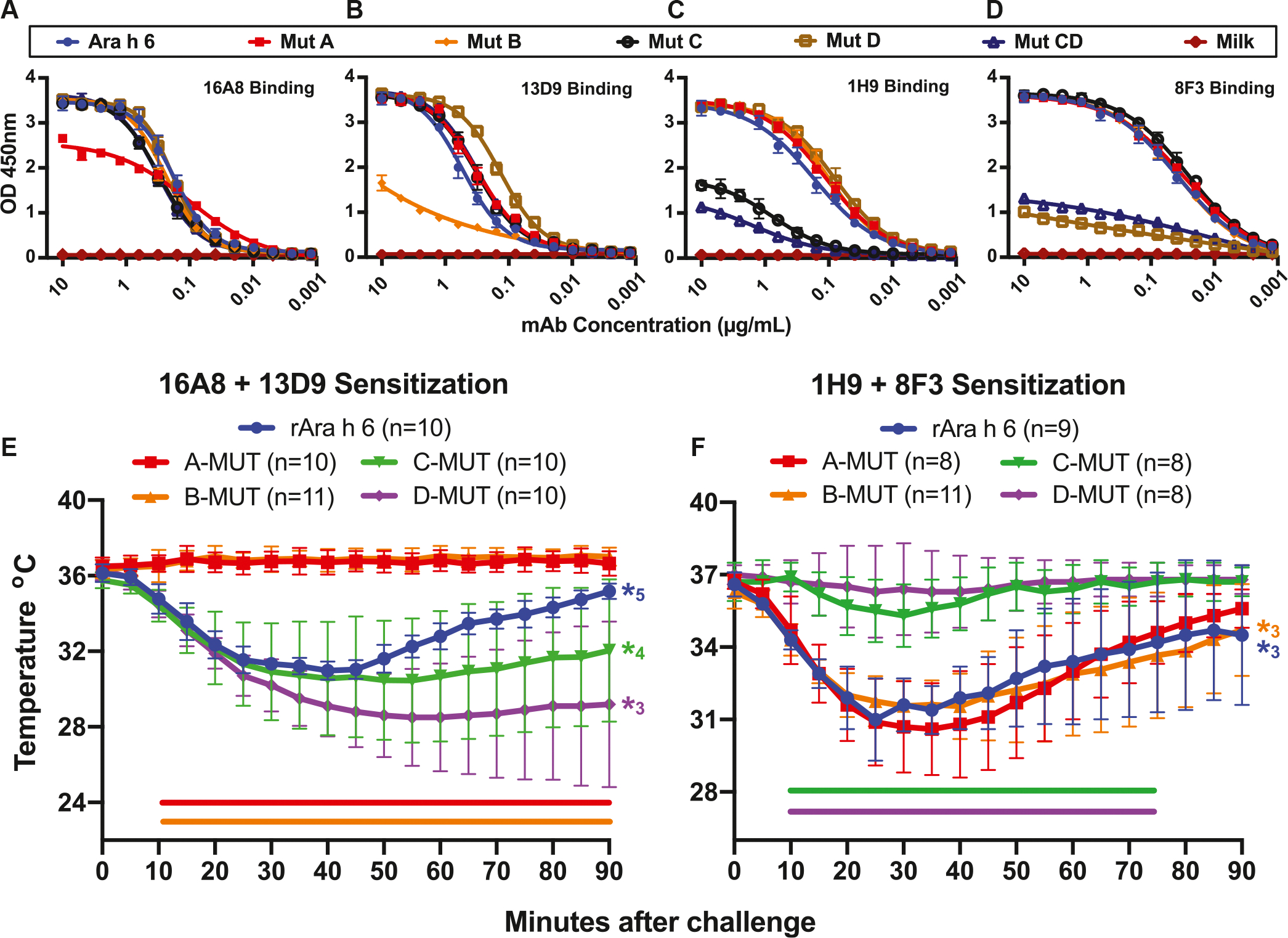
IgE binding to structure-based Ara h 6 mutant allergen proteins. Binding of **(A)** 16A8, **(B)** 13D9, **(C)** 1H9, **(D)** and 8F3 to mutant Ara h 6c allergen proteins. Human FCεRIa transgenic mice were sensitized with 100 μg total of human IgE **(E)** sites A and B and **(F)** sites C and D and challenged with mutant Ara h 6c allergen proteins. Anaphylaxis was monitored using an implanted temperature probe for 90 minutes following challenge. Time points with calculated *P* values < .05 are underscored with a colored bar. Data are means ± SD of each experimental group. The number of mice (n) for each experimental group is shown. The number of mice in each group that died as a result of anaphylaxis is indicated by an *asterisk*.

**FIG E1.**
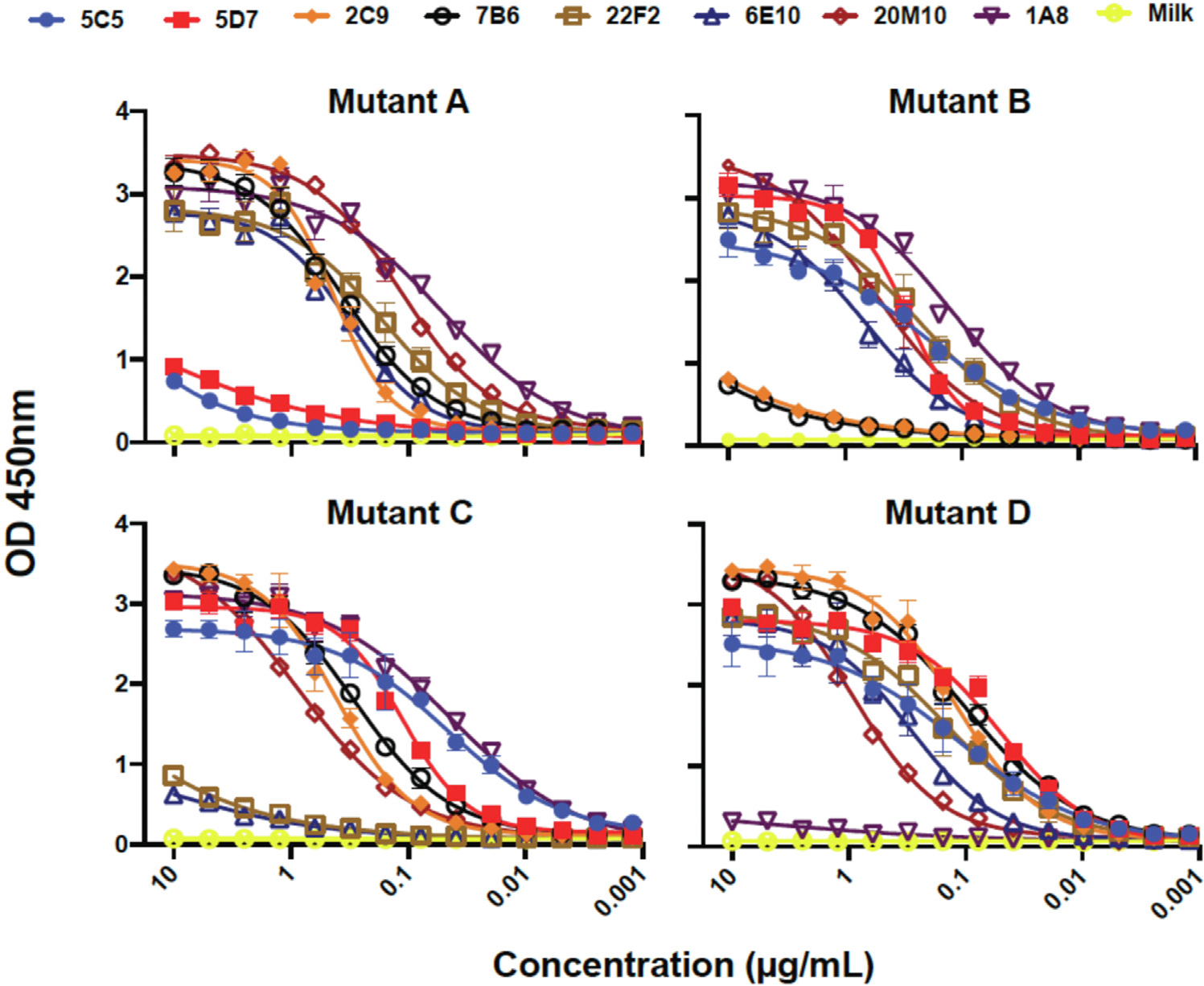
IgE binding to structure-based Ara h 6 mutant allergen proteins. Binding of 5C5 and 5D7 (site A), 2C9 and 7B6 (site B), 22F2 and 6E10 (site C), 1A8 and 20M10 (site D) to mutant Ara h 6 allergen proteins.

These mutations each severely disrupted recognition by the antibodies to which they were designed (16A8, 13D9, 1H9, and 8F3) without disrupting binding to antibodies recognizing nonmutated sites (Fig 6, A–D). Further, these mutated antigens also disrupted binding to additional antibodies that recognize the disrupted sites (Figure E1 in the Online Repository at www.jacionline.org). However, antibodies recognizing nonmutated sites were not affected, indicating gross structural defects were not introduced. Additionally, these mutant allergens did not induce anaphylaxis in our mouse model when challenged with antibodies against the mutated epitopes but did induce anaphylaxis when challenged with antibodies against nondisrupted epitopes (Fig 6, E and F).

## DISCUSSION

In this article, we focused on the critical contacts between allergens and naturally occurring IgE antibodies. Using x-ray crystallography, we defined the molecular interactions between peanut allergens and 5 pathogenic IgE antibodies identified from people with type I hypersensitivity reactions to peanut. Of the 5 antibodies described in detail here, 2 antibodies, 16A8 and 13D9, recognize both Ara h 2 and Ara h 6, and the epitopes for these antibodies are largely conserved between Ara h 2 and Ara h 6 (Fig 5). Antibodies 8F3 and 1H9 are Ara h 6 specific and bind epitopes that are not conserved on Ara h 2 (Fig 5). Antibody 38B7 is highly specific for Ara h 2 and binds a short peptide sequence DPYSP^OH^S that is unique to Ara h 2. This sequence motif is repeated twice in isoform Ara h 2.01 and 3 times in isoform Ara h 2.02. We anticipate that improved understanding of the epitopes relevant for disease in individual patients is possible using either a panel of blocking antibodies or a panel of hypoallergens to map the reactivity of patient sera. This would allow clinicians to observe changes in antibody titers against specific epitopes over time and to design treatment courses when targeted therapies become available. Here we have focused on Ara h 2 and Ara h 6 because of their importance, but we recognize the clinical relevance of other peanut allergens.^6–8^

The structures with 13D9, 8F3, and 1H9 all highlight the importance of conformational epitopes. These 4 antibodies recognize residues that are only in proximity to each other when the allergen is properly folded. 16A8 and 38B7, by contrast, recognize linear epitopes. In the case of 38B7, all important contacts are made to a linear epitope. Despite the presence of conformational epitopes, multiple studies have shown that heating via cooking does not eliminate recognition and may enhance it.^29–32^ These studies were done with polyclonal sera and with antigens that as well as having been heated, had also been SDS denatured, complicating a direct comparison to our structural studies. However, the presence of 3 38B7 epitopes in Ara h 2.02 and 2 epitopes on Ara h 2.01 suggest that different and larger immune complexes can form with 38B7 than with the site A, B, C, or D targeted antibodies. The importance of this epitope is further highlighted by a recent study showing both the prevalence of antibodies that recognize it and their ability to cause degranulation of human mast cells and anaphylaxis in humanized mice.^10^

The molecular details presented here represent examples of how specific mAbs recognize epitopes; whereas some features are likely to be conserved across many mAbs within a given bin, additional structures or reliable predictions are necessary to identify these conserved features. Importantly, the precise molecular details revealed by these structures enabled hypoallergen development, and these hypoallergens are not recognized by other members of the bin they target such that the designs built on a specific mAb are relevant for the entire bin. These hypoallergens are unable to elicit anaphylaxis in our mouse model both when sensitized with antibodies against which they were designed and when sensitized with other antibodies from the same bin. Intriguingly, recombinantly produced Ara h 2.01 or Ara h 2.02 is already a hypoallergen for 38B7 because it lacks the critical proline hydroxylation within the DPYSP^OH^S epitope. This suggests that modified allergen proteins deficient in their capacity to be bound by other antibodies will also be deficient in recognition by a common and important antibody class that is similar to 38B7.^10^ Hypoallergens to 1 or multiple antibody bins could become the basis of improved immunotherapy approaches in which the hypoallergen used first is determined by the patient’s serum reactivity.

The panel of IgE-derived antibody-antigen structures presented here can be compared with recent IgG-Ara h 2 complex structures that used IgGs from patients receiving peanut oral immunotherapy.^13,14^ In these studies, 3 epitope bins on Ara h 2 were described, and representative structures were determined. As shown in Fig E2 (in the Online Repository at www.jacionline.org), clone 22S1 recognizes the same epitope as 16A8 and would likely fall in our bin A. Clone 13T1 recognizes the same epitope as 13D9 and would likely fall into our bin B. The epitope bound by 13T5 is not represented in our panel of structures.

In addition to hypoallergen development, a panel of IgE-derived variable regions expressed as class switched human IgG_1_ has been developed and shown to inhibit anaphylaxis.^16^ Such reagents could be further developed to passively block the allergic reaction, thereby mitigating against the risk of anaphylaxis resulting from accidental exposure. Such reagents might also prevent the allergic or atopic march of worsening disease symptoms^33^ and increase the scope of immunotherapies.

Finally, the mechanism explained herein by which a single antibody clone can mediate anaphylaxis by binding a peptide repeat suggests a mechanism by which peanut allergy might arise. 38B7-like antibodies are functional without a second IgE to an additional site and may be initiators of peanut allergy. As additional antibodies develop that can pair with 38B7, allergy worsens, eventually spreading to homologous sites and then nonhomologous sites on Ara h 6.

## Figures and Tables

**FIG 2.**
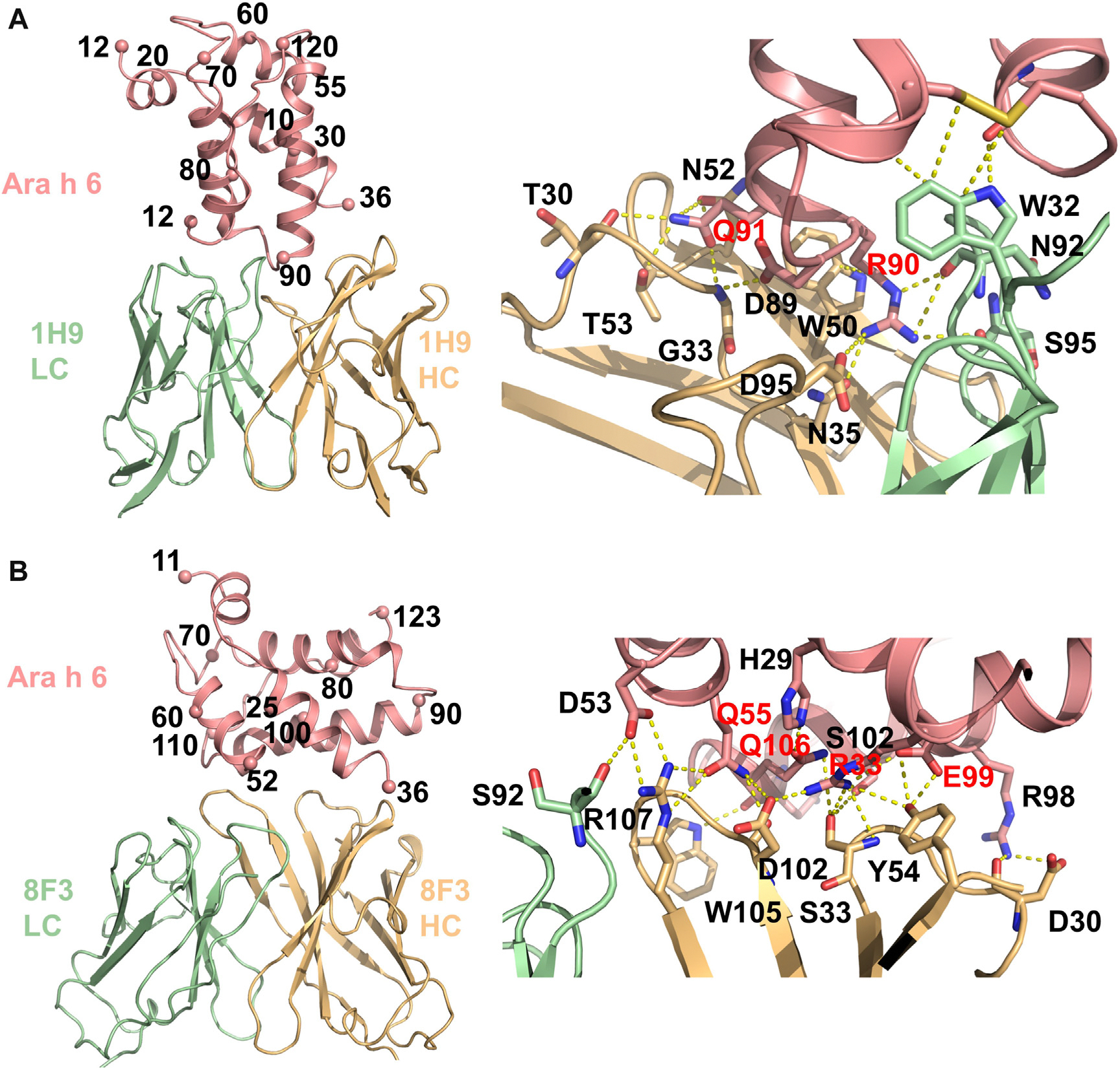
Structures of Ara h 6 specific clones, 1H9 and 8F3. Allergens are colored *salmon*, HCs are *light orange*, and LCs are *light green*. **(A)** Site C targeted antibody 1H9 recognizes the end of helix 4, the beginning of helix 5, the intervening loop, and the carboxy terminus of Ara h 6c *(left)*. This interface is approximately centered on key residues R90 and Q91 (labeled in *red*). Three H-bonds are formed between HC residues to Q91 (from T30_HC_, G33_HC_, and T53_HC_), and R90 is recognized with a salt bridge from D95_HC_ and H-bonds from N35_HC_, N92_LC_, and S95_LC_. In addition, a hydrophobic pocket on Ara h 6c is filled by W32_LC_, which also H-bonds to G121. **(B)** Site D residues R33, Q55, E99, and Q106 are recognized by the HC of 8F3. These residues are buried, in part by H29, and involved in multiple polar interactions. R33 salt bridges with D102 and H-bonds with S33 and Y54. Q55 H-bonds with R107_HC_ and D102_HC_. E99 forms an H-bond to Y54. Q106 H-bonds with S33_HC_. In the middle of the interface, S102 H-bonds to S33_HC_. Two peripheral HC interactions include a salt bridge and H-bonds between R98 and D30_HC_ and an H-bond between the indole nitrogen of W105_HC_ and the main chain of Q105. D53 salt bridges to R107_HC_ and the main chain of S92_LC_.

**FIG 2.**
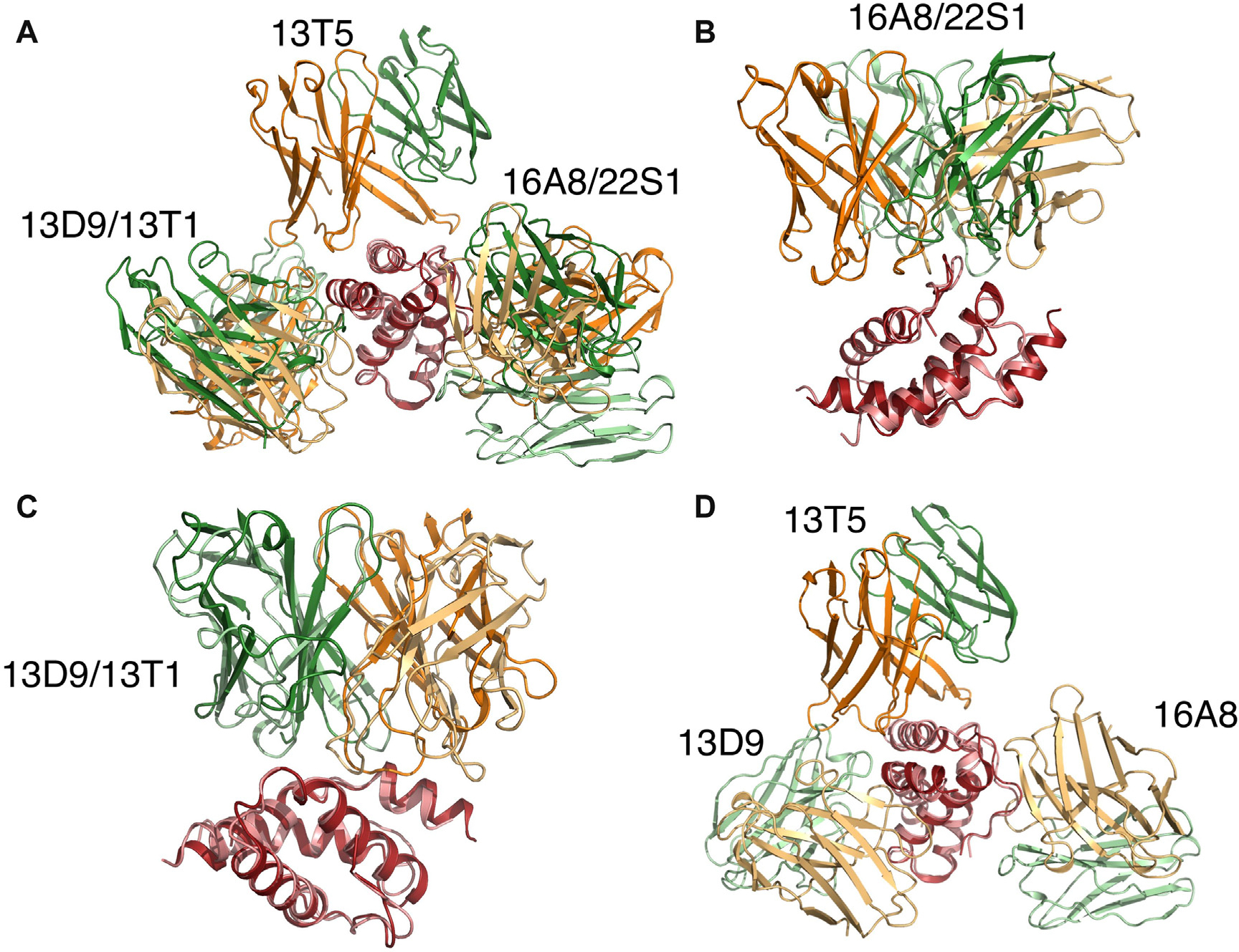
Comparison to IgG-Ara h 2 structures. Two recent PDB entries, 8DB4 and 8GP4, present structures of 3 human IgG_4_-derived Fabs bound to Ara h 2: 22S1, 13T1, and 13T5.^13,14^ 22S1 recognizes site A, 13T1 recognizes site B, and 13T5 recognizes a unique epitope. Ara h 6c is colored *salmon*, Ara h 2 is *red*, IgE-derived HCs are light orange, IgG_4_-derived HCs are orange, IgE-derived LCs are *light green*, and IgG_4_-derived LCs are *dark green*. Structural alignments were done on the allergens. Only variable regions of the antibodies are shown. **(A)** Overlay of all structures. **(B)** Overlay of 16A8 and 22S1, showing that both antibodies recognize site A. The antibodies are in completely different orientations. **(C)** Overlay of 13D9 and 13T1. Both recognize site B, and the antibodies themselves approximately overlap. **(D)** Overlay of 16A8, 13D9, and 13T5. 13T5 recognizes a unique epitope that is not represented by any of the structures presented in this article.

**TABLE I.** Human IgE mAb kinetic analyses by surface plasmon resonance

	Ligand	n	Analyte									
			Mean *k*_a_ (M^−1^s^−1^)	*k*_a_ SD	Mean *k*_d_ (s^−1^)	*k*_d_ SD	Mean *K*_D_ (M)	*K*_D_ SD	Mean Rmax (RU)	Rmax SD	Mean res sd	SD res sd

nArah2	38B7	6	2.96 × 10^5^	1.66 × 10^4^	2.90 × 10^−4^	2.19 × 10^−5^	9.80 × 10^−10^	9.30 × 10^−11^	138.7	23.5	6.9	1
rArah2.01		6	1.55 × 10^4^	1.63 × 10^3^	4.59 × 10^−4^	6.23 × 10^−5^	3.00 × 10^−8^	5.10 × 10^−9^	47.4	11.2	2.4	0.39
rArah2.02		6	1.65 × 10^5^	6.04 × 10^4^	4.74 × 10^−4^	1.43 × 10^−5^	3.50 × 10^−9^	1.30 × 10^−9^	177.1	35.5	13	2.3
rArah2_loopdel			No binding									
38B7pep_std		2	1.69 × 10^4^	9.46 × 10^3^	8.87 × 10^−2^	1.03 × 10^−2^	7.10 × 10^−6^	4.10 × 10^−6^	43.7	16.6	2.7	0.56
38B7pep_OH		8	1.01 × 10^5^	2.48 × 10^4^	1.98 × 10^−3^	4.30 × 10^−4^	2.00 × 10^−8^	6.50 × 10^−9^	89	21.7	3.9	0.58
16A8pep_std			No binding									
Random_std			No binding									
rArah6			No binding									
nArah2	16A8	8	1.06 × 10^5^	2.75 × 10^4^	2.90 × 10^−4^	3.78 × 10^−5^	3.00 × 10^−9^	8.60 × 10^−10^	63.6	8.9	3.8	0.66
rArah2.01		6	8.72 × 10^5^	6.54 × 10^4^	5.63 × 10^−5^	8.82 × 10^−6^	6.50 × 10^−11^	1.10 × 10^−11^	212	54.9	11	2.4
rArah2.02		4	1.35 × 10^6^	6.05 × 10^5^	3.82 × 10^−5^	7.40 × 10^−6^	3.60 × 10^−11^	1.70 × 10^−11^	106	59.9	4.7	2.2
rArah2_loopdel		2	2.22 × 10^6^	5.64 × 10^4^	3.69 × 10^−5^	4.76 × 10^−7^	1.70 × 10^−11^	4.70 × 10^−13^	119.7	1.4	7.6	0.84
16A8pep_std		2	2.63 × 10^8^	8.70 × 10^7^	2.90 × 10^2^	1.39 × 10^2^	1.00 × 10^−6^	6.10 × 10^−7^	14.9	8.90 × 10^−4^	2.3	0.12
38B7pep_OH			No binding									
Random_std			No binding									
Arah6		2	2.39 × 10^6^	1.06 × 10^4^	8.57 × 10^−5^	5.10 × 10^−6^	3.60 × 10^−11^	2.10 × 10^−12^	117.1	3.7	7.0	1.2

Kinetic parameters of 38B7 and 16A8 antibody binding to Ara h 2 variants and peptide epitopes using a Carterra LSA instrument are shown. The number of analyte concentrations used in the analysis (n), K_a_, dissociation rate constant K_d_, and K_D_ are shown for each set of experiments.

*K_a_*, Association rate constant; *K_d_*, dissociation rate constant; *K_D_*, dissociation equilibrium constant; *res sd*, residual standard deviation; *Rmax*, maximal feasible surface plasmon resonance signal.

**TABLE E1.** Data collection and refinement statistics

	38B7	38B7_PepOH	AraH2-8F3-38B7	AraH6-16A8	AraH6-8F3-1H9	AraH6-13D9-16A8

PDB accession code	8SHV	8SHW	8SJ6	8SI1	8SJ4	8SJA
Resolution	28.4-1.58 (1.61-1.58)	30.4-1.95 (2.02-1.95)	97.1-3.97 (4.11-3.97)	52.8-3.2 (3.31-3.2)	55.0-2.67 (2.77-2.67)	44.8-2.69 (2.77-2.69)
Space group	P 2_1_ 2_1_ 2_1_	C 2	P 2 2_1_ 2_1_	C 2 2 2_1_	P 2_1_	P 2_1_ 2_1_ 2_1_
Unit cell (Å^2^)	62.0, 65.8, 136.7	128.0, 91.8, 83.0	44.4, 182.9, 194.2	105.6, 155.3, 286.4	39.4, 85.4, 165.8	108.5, 112.1, 223.2
		β = 93.2°			β = 96.2°	
Total reflections	568,062 (22,210)	289,732 (29,262)	57,338 (5,774)	164,161 (17,916)	17,1209 (11,767)	34,5718 (36,162)
Unique reflections	77,127 (3,660)	67,997 (6,725)	14,122 (1,392)	36,312 (3,861)	30,930 (2,898)	75,706 (7,535)
Multiplicity	7.4 (6.1)	4.3 (4.4)	4.1 (4.1)	4.5 (4.6)	5.5 (4.0)	4.6 (4.8)
Completeness (%)	99.8 (96.2)	97.6 (96.6)	97.5 (98.7)	92.5 (100)	99.3 (93.6)	99.3 (100)
Mean I/sigma(I)	12.7 (1.0)	15.6 (2.4)	10.2 (2.3)	20.7 (2.4)	13.0 (2.4)	15.5 (2.4)
Wilson B factor	25	34	120	104	43	67
R merge	0.070 (1.406)	0.050 (0.65)	0.123 (0.652)	0.051 (0.671)	0.116 (0.643)	0.063 (0.703)
CC1/2	0.999 (0.406)	0.999 (0.837)	0.996 (0.799)	0.999 (0.798)	0.992 (0.66)	0.999 (0.824)
Reflections used in refinement	77,039 (7,466)	67,972 (6,718)	14,106 (1,392)	36,299 (3,860)	30,916 (2,898)	75,689 (7,534)
Reflections used for Rfree	3,735 (366)	3,336 (370)	670 (78)	1,735 (189)	1,517 (124)	3,897 (419)
RWork	0.164 (0.325)	0.178 (0.255)	0.252 (0.341)	0.247 (0.375)	0.191 (0.287)	0.200 (0.332)
Rfree	0.178 (0.338)	0.205 (0.304)	0.301 (0.445)	0.300 (0.427)	0.233 (0.373)	0.249 (0.381)
No. non-hydrogen atoms	3,851	7,474	7,501	11,439	7,429	14,636
Protein atoms	3,347	6,759	7,501	11,439	7,266	14,515
Ligands	32	64	0	0	0	14
Solvent	489	651	0	0	163	113
Protein residues	431	876	969	1,490	950	1,907
RMS (bonds)	0.013	0.003	0.002	0.002	0.003	0.003
RMS (angles)	1.15	0.59	0.45	0.51	0.55	0.65
Ramachandran favored (%)	98.12	97.78	96.53	96.12	97.32	94.3
Ramachandran allowed (%)	1.88	2.22	3.36	3.74	2.68	4.7
Ramachandran outliers (%)	0.0	0.0	0.11	0.14	0.0	1.0
Mean B factor (Å^2^)	32	47	145	135	59	81
Protein	31	47	145	135	59	81
Ligands	36	62	—	—	—	83
Solvent	42	46	—	—	40	60

Statistics for the highest-resolution shell are shown in parentheses.

*PDB*, Protein Data Bank; *RMS*, root mean square.

## Key Messages

- Two common antigenic sites, sites A and B, are shared between Ara h 2 and Ara h 6.
- Three common sites are conformational and present only when the allergens are properly folded.
- Antibodies against a repeated linear epitope can induce anaphylaxis without a second site being engaged.

## Abbreviations used

CDR: Complementarity determining region
Fab: Antigen-binding fragment
H-bond: Hydrogen bond
HC: Heavy chain
LC: Light chain
PEG: Polyethylene glycol
